# Food in Health and Disease

**Published:** 1890-03

**Authors:** 


*Food in Health and Disease.* By I. BURNEY YEO, M.D., F.R.C.P. London: CASSELL & CO., LIMITED. 1889.

Cassell's series of Clinical Manuals would not have been complete without a work dealing with this subject; and if this volume does not add much to the information given by existing treatises, yet it is much to be commended for the excellent arrangement of its contents, which, under the two main heads of the title, are subdivided into all necessary detail. It seems inevitable that writers on Food should consider the human stomach to be a kind of retort in which chemical changes go on with unerring accuracy; and so Dr. Yeo, in the first part of his book, gives plenty of analytical tables to guide in the preparation of a normal dietary. Without these it seems almost a marvel that instinct should ever have led healthy men to choose food that in fit proportions would properly nourish them. The second and smaller part of Dr. Yeo's book, dealing with Food in Disease, is of principal value; and here, with the exception of an over-estimate of the food-value of beef-tea and its allies, will be found much that is helpful to the practical medical man.

This book is turned out in the flimsy and shabby way which Messrs. Cassell and Company seem to think good enough for their medical publications.

